# Effect of Tephrosia purpurea (L.) Pers. Leaves on Gentamicin-Induced Nephrotoxicity in Rats

**DOI:** 10.3797/scipharm.1302-09

**Published:** 2013-07-22

**Authors:** Avijeet Jain, Alok Nahata, Abhay Kumar Singhai

**Affiliations:** 1 Department of Pharmaceutical Sciences, Doctor Hari Singh Gour Vishwavidyalaya, Sagar-470003 (M.P.), India.; 2 Mittal Institute of Pharmacy, Opp. BMHRC, Navibagh, Bhopal-462038 (M.P.), India.

**Keywords:** Blood urea, Serum creatinine, Nephroprotective, Quercetin, Gentamicin

## Abstract

The aim of the study was to evaluate the nephroprotective and nephrocurative effects of *Tephrosia purpurea* (L.) Pers. leaves against gentamicin-induced acute renal injury in albino rats. The maximum free radical scavenging activity of the ethanolic extract was the basis for the selection of this extract for the *in vivo* study. Gentamicin (40 mg/kg, s.c.) was administered to induce toxicity in the toxic group and the ethanolic extract (200 mg/kg p.o.) was administered in all treated groups. Blood urea and serum creatinine levels were monitored to assess the effects. The antioxidant potential was also evaluated by the estimation of reduced glutathione (GSH) and malondialdehyde (MDA). Gentamicin intoxication caused significant increases in blood urea and serum creatinine levels as compared to the normal control. In the preventive regimen, the extract (200 mg/kg, p.o.) showed significant reductions in the elevated blood urea and serum creatinine. Histopathological changes were in accordance with the biochemical findings. Also in the curative regimen, the blood urea and serum creatinine levels revealed significant curative effects. In our *in vivo* antioxidant activity, the GSH level was significantly (P< 0.05) increased in the extract-treated groups, whereas MDA was reduced significantly (P< 0.05). Further thin layer chromatography (TLC) and high-performance thin layer chromatography (HPTLC) led us to ascertain the presence of rutin and quercetin in the extract. We were able to isolate and characterize an isolate from the ethanolic extract and characterize it on the basis of chromatographic, melting point, FTIR, NMR, and mass spectroscopic studies. The findings suggest that the ethanol extract of *Tephrosia purpurea* leaves possesses marked nephroprotective and curative activities without any toxicity. The proposed mechanisms for the claimed activity are antioxidant activity and the inhibition of an overproduction of NO and Cox-2 expression. These activities may be attributed to the presence of phenolics and flavonoidal compounds like rutin and quercetin. Thus, it can be said that *Tephrosia purpurea* could offer a promising role in the treatment of acute renal injury caused by nephrotoxins like gentamicin.

## Introduction

*Tephrosia purpurea* (L.) Pers. (Fabaceae) (TP), commonly known in Sanskrit as ‘Sarapunkha’, is a highly branched, sub-erect, herbaceous perennial herb [1]. According to Ayurvedic literature, this plant is also given the name of ‘wranvishapaka’ which means that it has the property of healing all types of wounds [2]. It is an important component of some preparations such as Tephroli and Yakrifit used for liver disorders [3, 4]. In the Ayurvedic system of medicine, various parts of this plant are used as a remedy for impotency, asthma, diarrhea, gonorrhea, rheumatism, ulcers, and urinary disorders. The plant has been claimed to cure diseases of the kidney, liver, spleen, heart, and blood [2, 5]. The dried herb is effective as a tonic laxative, diuretic, and deobstruent. It is also used in the treatment of bronchitis, bilious febrile attack, boils, pimples, and bleeding piles. The roots and seeds are reported to have insecticidal and piscicidal properties, which are also used as vermifuge. The roots are also reported to be effective in leprous wounds and the juice in skin eruptions. An extract of the pods is effective for pain and inflammation, and their decoction is used against vomiting [6]. The ethanolic extract of the seeds has shown significant *in vivo* hypoglycaemic activity in diabetic rabbits [7]. The ethanolic extracts of TP possess potential antibacterial activity. The flavonoids were found to have antimicrobial activity [8]. Phytochemical investigations on TP have revealed the presence of glycosides, rotenoids, isoflavones, flavanones, chalcones, flavanols, and sterols [9].

Acute renal failure refers to the sudden and usually reversible loss of renal function, which develops over a period of days or weeks. Among the causes of acute renal failure, acute tubular necrosis, which occurs due to ischemia or nephrotoxins like cisplatin and gentamicin, is most common, accounting for 85% of the incidence. Gentamicin, an amino-glycoside antibiotic, is used as an effective agent against Gram-negative infections. Its chemical stability and rapid bactericidal action has made it a first-line drug in a variety of clinical situations. However, nephrotoxicity is the major side effect of aminoglycosides accounting for 10–15% of all cases of acute renal failure [10]. Studies have also shown that 30% of the patients treated with gentamicin for more than seven days show signs of nephrotoxicity [11]. It has been shown that the specificity of gentamicin renal toxicity is related to its preferential accumulation in the renal convoluted tubules and lysosomes [12].

There is a continuous search for agents which provide nephroprotection against the renal impairment caused by drugs like cisplatin and gentamicin, for which allopathy offers no remedial measures. Thus, it is imperative that mankind turns towards alternative systems of medicine for treatment. Hence, the present study is an attempt to screen TP leaves for their nephroprotective and curative activities.

In the ethnobotanical claims, TP is used for the treatment of renal diseases. To the best of our knowledge, there is no scientific report available in support of the nephroprotective activity of TP leaves. Therefore, to justify the traditional claims we have assessed the nephroprotective and curative effect of TP leaves using gentamicin-intoxicated rats. Free radical scavenging and *in vivo* antioxidant activities were also determined. Ethanolic extract with maximum free radical scavenging activity was selected for the *in vivo* studies.

## Experimental

### Plant Collection

TP was collected from the forests around Dr. H.S. Gour Vishwavidyalaya, Sagar in September 2006 and were identified at the Department of Botany, Dr. H.S. Gour Vishwavidyalaya, Sagar (Voucher specimen no. TP/AJ/3003).

### Preparation of Extracts

TP dried leaves were coarsely powdered and defatted with petroleum ether in a soxhlet extractor until complete extraction. The defatted drug was successively extracted with chloroform, ethyl acetate, and ethanol in soxhlet extractors to get the respective extracts. Water extract was prepared by maceration of the marc leftover after the successive extraction processes. Percentage yield was calculated for each extract after drying under a vacuum. The percentage yield of chloroform, ethanol, ethyl acetate, and water extract were 4.1, 5.7, 10.14, and 8.9%, respectively.

### Phytochemical Screening

All the extracts were subjected to phytochemical screening according to standard chemical tests for the presence of various phytoconstituents. The phytochemical studies have shown the presence of flavonoids, glycosides, phenolic compounds, alkaloids, carbohydrates, proteins, saponins, and amino acids

### Chromatographic Profiling of TP using TLC and HPTLC Studies

The ethanolic extract showed a positive Shinoda test indicating the presence of flavonoids. Co-chromatography with the standard flavonoidal compounds, i.e. rutin and quercetin, on the thin layer chromatographic plates (TLC) showed the spots at the same Rf as the standard compounds, further clarifying the presence of flavonoidal compounds in the extract. The solvent system used for TLC was methanol: water: formic acid (40:57:3) and Rf observed for quercetin and rutin was 0.07 and 0.17, respectively.

TLC aluminum plates pre-coated with silica gel RP-18_F254_ S were used. High-performance thin layer chromatographic (HPTLC) densitometric determination of these compounds was carried out at 254 nm with the same solvent system used for TLC.

**Fig. 1:**
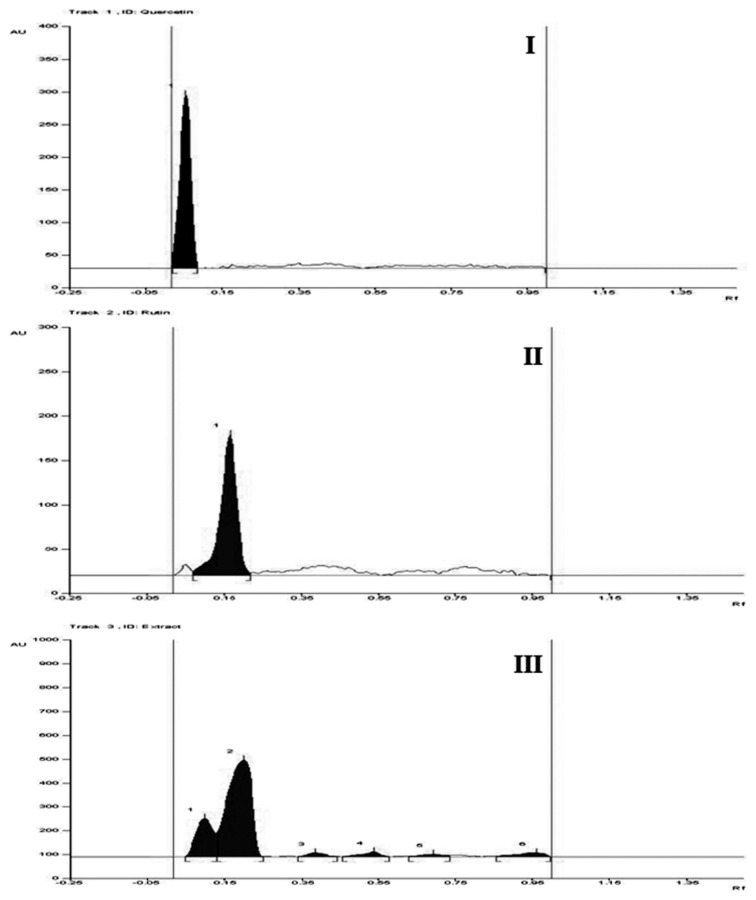
HPTLC Densitogram of quercetin (I), rutin (II), and TP ethanolic extract (III) at 254 nm

The solvent system produced good separation with Rf values of 0.07 (quercetin) and 0.17 (rutin). The ethanolic extract resolved into six compounds in the developing solvent system. The identity of the bands of quercetin and rutin in the ethanolic extract was confirmed by comparing the HPTLC densitogram of the extract with those of standards using a Camag HPTLC system equipped with an automatic sample application device (Linomat 5) and a TLC scanner 3 (WINCATS version 1.2.3) with a UV cabinet (Fig. 1). The quantification of rutin and quercetin was done and the percentage of both the compounds was reported to be 0.473 and 1.913%, respectively, for quercetin and rutin, respectively. This has been reported in one of our previous publications [13].

### Isolation of the Compound from TP Leaves and its Characterization

The isolation of the compound from the ethanolic extract of TP leaves was carried out on the basis of solubility [14]. For isolation, distilled water (100 ml) was added to the concentrated ethanolic extract (50 ml). After about 1 h, precipitation was observed. This precipitate was recovered by filtration. Furthermore, the precipitate was dissolved in chloroform (100 ml) by shaking for 15 min and heated gently for 5 min and filtered in a hot state. The chloroform soluble fraction was discarded, and the insoluble fraction left on the filter paper was dissolved in ethyl acetate (100 ml) by shaking for 15 min, heated gently for 5 minutes, and filtered in a hot state. The ethyl acetate soluble fraction was discarded, and the insoluble fraction left on filter paper was crystallized with methanol. Thereafter, the residue obtained was subjected for qualitative, chromatographic, and spectral studies. The isolate was designated as compound 1.

Co-TLC with standard rutin and quercetin indicated the presence of rutin as an isolated compound. The confirmation of the structure was done using melting point, FTIR, NMR, and mass spectroscopy.

Compound 1, melting point (m.p.) 180–184 °C (using superfit melting point apparatus), buff in color has shown strong absorption bands at 3415 (-OH) 1602.1 (unsaturated C=O), 2933 (C-Me), 1060 cm^−1^(glycosidic C–O) in FTIR spectra (KBr pellet method). The instrument was from GBC Cintra, Australia. The NMR spectra were measured in DMSO (Bruker Avance 400 II NMR). Chemical shifts were given in δ values relative to TMS. NMR spectroscopy showed a peak at 6.21 (1H, d, J=2, C6-H), 6.40 (1H, d, J=2, C8-H), 7.55 (1H, d, J=2.1,C2′-H), 6.86 (1H, d J=9,C5′-H), 7.56 (1H, dd, J=9,2.1, C6′-H), 9.71 (1H, s, C4′-OH), 9.21 (1H, s, C3′-OH), 12.62 (1H, s, C5-OH), 10.86 (1H, s, C7-OH), 5.35 (1H, d, J=7.4, H1-G), 5.12 (1H, d, J=1.9, H1-R), 1.00 (3H, d, J=6.1,CH3-R). (R and G represent signals from rhamnose and glucose moieties, respectively). The mass spectrum has shown fragmentation of the compound, with a base peak at m/z 610 with other prominent peaks at 463, 447, 301, 179, and 151. Based on the spectral studies, compound 1 was found to be rutin.

Although we were not able to isolate quercetin, we could quantify the presence of both the compounds in the ethanolic extract and concluded that quercetin and rutin were present in the proportions of 0.473 mg/100 mg and 1.913 mg/100mg, respectively. This has been discussed in the section above.

### Animals

Healthy adult albino rats (100–150 g) of both sexes aged 60–90 days were used for the study. The rats were housed in polypropylene cages and maintained under standard conditions (12-h light: 12-h dark cycle; 25±3 °C; 35–60% humidity). The animals had free access to standard lab chow (Hindustan Lever Ltd., Mumbai, India) and water *ad libitum*. The study was conducted after obtaining institutional animal ethical committee clearance (412/01/ab/CPCSEA, India).

### Acute Toxicity Studies

The ethanolic extract was suspended in gum acacia (2% w/v) and administered to the animals in increasing dosage levels. The dose of the extract was calculated as 1/10th of the maximum tolerated dose (2000 mg/kg b.w.) [15].

### Superoxide Free Radical Scavenging Activity

This was determined by the NBT (nitro blue tetrazolium) reduction method. The assay was based on the capacity of the sample to inhibit blue formazan formation by scavenging the superoxide radicals generated in the riboflavin-light-NBT system. The reaction mixture contained EDTA, riboflavin, NBT, various concentrations of extracts (1–100 μg/ml), and phosphate buffer pH (7.6) in a final volume of 3 ml. The tubes were uniformly illuminated with an incandescent lamp for 15 min and absorbance was measured at 590 nm before and after illumination. The percentage inhibition of superoxide generation was measured by comparing the absorbance values of the control with those of the test [16].

### DPPH Free Radical Scavenging Activity

A stock solution of 0.1 ml of DPPH was prepared in ethanol. This solution was mixed with equal volume of solutions (different concentrations) of the test extract. The reaction was allowed to complete in the dark for about 20 minutes. The absorbance was taken at 517 nm. The experiment was repeated three times. The difference in absorbances between the test and the control was calculated and expressed as percentage scavenging of the DPPH radical [17].

### Total Antioxidant Activity

The total antioxidant activity of all the extracts was measured in concentrations of 10 –1000 μg/ml [16]. Briefly, peroxidase (4.4 unit/ml, 0.2 ml), H_2_O_2_ (50 mM, 0.2 ml), ABTS [2,2′-azino-bis(3-ethylbenzothiazoline-6-sulfonic acid), 100 mM, 0.2 ml], and distilled water (1.4 ml) were mixed and kept in the dark for 1 h to form a bluish-green complex. After adding the various concentrations of the tested extracts (1 ml), the absorbance at 734 nm was measured.

### Gentamicin-Induced Renal Damage

The test samples were prepared in the distilled water. A weighed quantity of the ethanolic extract was suspended in distilled water by trituration. The extract was taken in a pastel motor and triturated continuously to get a homogenous suspension. The suspension was stored in an airtight bottle in a cool and dry place.

The animals were divided into four groups of six rats each and all testing drugs were administered orally at the calculated dose:

Group I: control to which neither drug nor gentamicin was administered
Group II: treated with only gentamicin (40 mg/kg) s.c.
Group III (preventive): treated with an ethanolic extract suspension (200 mg/kg/day) p.o. 30 minutes before gentamicin administration.
Group IV (curative): treated with an ethanolic extract suspension (200 mg/kg/day) p.o. 7 days after gentamicin administration.

In the preventive groups, the dose of gentamicin and extracts were given once a day, whereas in the curative regimen, gentamicin was administered twice a day. After 16 days, the animals were sacrificed by cervical dislocation and blood samples were collected from the inferior vena cava. To get the serum from the blood samples, the freshly drawn blood was centrifuged at 2500 rpm for 30 minutes and placed in a refrigerator.

### Biochemical Determinations

Blood urea concentration in the blood was estimated by an enzymatic method using a urease enzyme kit by the modified Berth Elot method [18]. Absorbance was read using a UV-240 Vis spectrophotometer (Shimadzu Corporation, Japan). Serum creatinine levels in serum was estimated by the alkaline picrate method using a creatinine kit [19]. Absorbance was read by a UV-240 Vis spectrophotometer.

### In vivo Antioxidant Activity

Reduced glutathione (GSH) and malondialdehyde (MDA) were estimated to assess *in vivo* antioxidant activity. The methods of Ellamn Georg [20] with some modification and Uchiyama and Mihara [21] were used, respectively.

### Histopathological Examination

Animals from each group were sacrificed on the day of blood withdrawal and the kidneys were isolated. Tissue samples were immersed in 10% formalin for the histopathological studies. The samples were embedded in paraffin, sectioned, and stained with haematoxylin and eosin. Blinding was employed in the study during the analysis of the slides, and the evaluator was kept blind regarding the nature of the slides and corresponding groupings.

### Statistical Analysis

Results are expressed as mean±SEM. Data were analyzed using one-way ANOVA followed by the Dunnett’s test. P<0.05 was considered to be statistically significant.

## Results

### Phytochemical Study

All the extracts were subjected to phytochemical studies (Table 1). The ethanolic extract showed a positive test for flavonoids, glycosides, phenolic compounds, alkaloids, carbohydrates, and amino acids etc.

### Chromatographic Profiling of TP using TLC and HPTLC Studies

The ethanolic extract showed a positive Shinoda test indicating the presence of flavonoids. Co-chromatography with standard flavonoidal compounds, i.e. rutin and quercetin, on TLC showed the spots at the same Rf as the standard compounds, further clarifying the presence of flavonoidal compounds in the extract. The densitometric HPTLC profile for the standards and the ethanolic extract is shown in figure 1. The quantification of rutin and quercetin was done and the percentage of both of the compounds was reported to be 1.913 and 0.473%, respectively.

### Isolation of the Compound from TP Leaves and its Characterization

Isolation of the compound from the ethanolic extract of TP leaves was carried out on the basis of solubility [14]. Co-TLC with standard rutin and quercetin indicated the presence of rutin as an isolated compound. The confirmation of the structure was done using melting point, FTIR, NMR, and mass spectroscopy. After all the analytical and structural confirmations using the above-mentioned methods, the isolated compound was found to be rutin, a rhamno-glucoside of quercetin. As it was isolated in a very low yield which was sufficient only for its structural characterization, it could not be included in our *in vivo* studies. But it could be considered as a biological and phytochemical marker on the basis of the results of the *in vivo* studies which have been discussed in the sections below.

### Superoxide Scavenging Activity

**Fig. 2:**
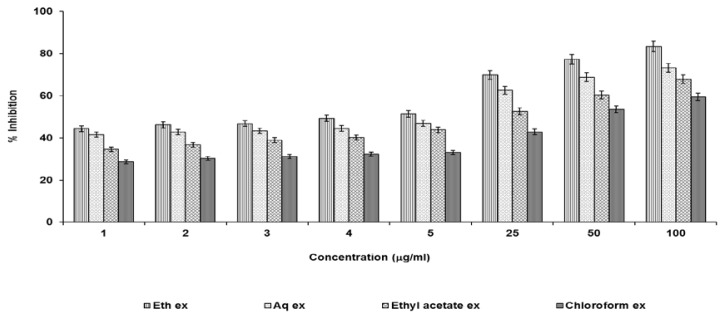
Superoxide free radical scavenging activity of test extracts

The NBT method was applied for the screening of the superoxide free radical scavenging activity. All the extracts were screened in the concentration of 1–100 μg/ml. The ethanolic extract showed a maximum percent reduction followed by the aqueous, ethyl acetate, and chloroform extracts. This activity was dose-dependent in all cases (Figure 2).

### DPPH Free Radical Scavenging Activity

**Fig. 3:**
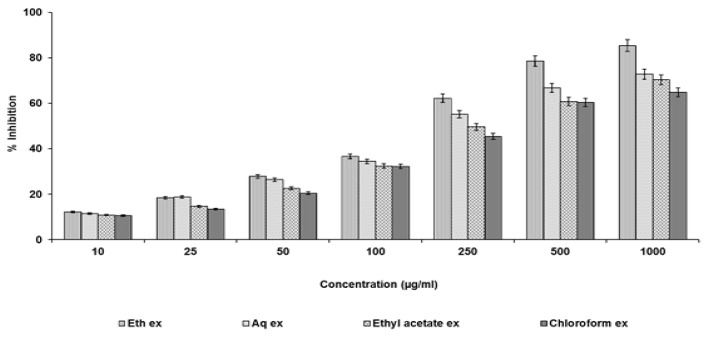
DPPH free radical scavenging activity of test extracts

Figure 3 shows the results of the activity. Ethanolic extracts at 1000 μg/ml showed maximum inhibition. Other extracts showed the results in a similar pattern.

### Total Antioxidant Activity

**Fig. 4:**
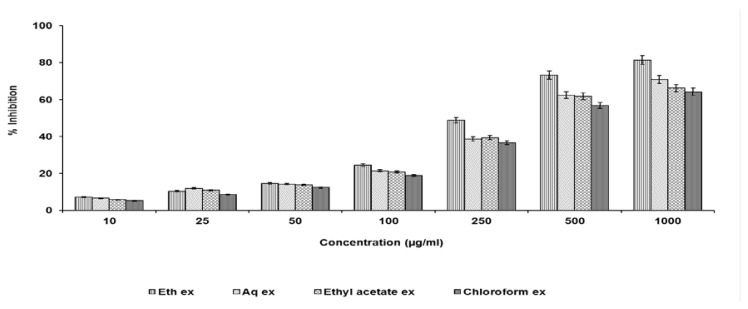
Total antioxidant activity of test extracts

All the extracts were screened in concentrations of 10–1000 μg/ml (Figure 4). The ethanolic extract showed maximum inhibition amongst all the extracts.

*In vitro* antioxidant activity screening was made as the basis for the selection of the extract for further studies. Results revealed that the ethanolic extract had the best activity and hence, was subjected to the *in vivo* studies.

### Acute Toxicity Studies

The acute toxicity of the extract was determined in albino mice. The animals were fasted overnight prior to the experiment. The animals were divided into eight groups. The extract was administered orally to various groups of mice in doses ranging from 1000, 1200, 1400, 1600, 1800, 2000, 2200, and 2500 mg/kg for the acute toxicity study [15]. There was no lethality in any of the groups after seven days of treatment.

### Gentamicin-Induced Renal Damage

Gentamicin-induced renal injury was evidenced by the elevated blood urea and serum creatinine levels. Table 2 shows the results of the biochemical estimation in the various groups, where blood urea and serum creatinine estimations express the nephroprotective and curative activities of the ethanolic extract. The study revealed increased levels of blood urea (69.48 ± 4.34 mg/dl) and serum creatinine (3.017 ± 0.208 mg/dl) in gentamicin-intoxicated animals as compared to the control which showed levels of 33.72 ± 1.92 and 0.818 ± 0.073 mg/dl, respectively. On the other hand, it was observed that 16 days of administration of the extract (200 mg/kg, p.o.), prior to gentamicin administration (40 mg/kg, s.c. single dose) in the prophylactic regimen, provided marked protection against gentamicin-induced renal injury. Similarly, in the curative regimen, the ethanol extract recovered the renal damage induced by gentamicin, suggesting its nephrocurative activity in gentamicin models of renal injury. The histopathological studies also supported these results.

### In vivo Antioxidant Activity

Activity was assessed by the estimation of reduced glutathione (GSH) and malondialdehyde (MDA). Results are cited in Table 3. Results of the study clearly revealed an increase in the levels of MDA in the gentamicin-intoxicated rats (462 ± 4.3 nmol/mg protein) compared to the control group (197.7 ± 2.1 nmol/mg protein). Treatment with the extracts significantly prevented the increase in these levels. GSH significantly increased in the extract-treated groups, whereas the gentamicin-intoxicated group showed a significant decrease in levels compared to the control group. The curative groups showed better results.

### Histopathological Studies

**Fig. 5:**
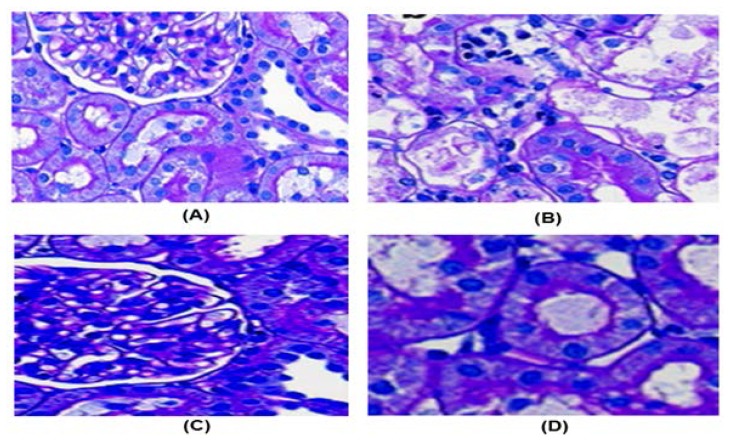
Histopathological observations. (A) Control group: normal kidney structure in which normal glomeruli with Bowmen’s capsule was seen. No tubular damage was seen. (B) Gentamicin-intoxicated group: kidney is severely damaged. Renal tubules are damaged with breaking up of tubular lining. Glomerular capsule shows degenerative changes. Tubular cast was found due to the breakdown of RBC of the glomeruli. (C) *Tephrosia purpurea* (200 mg/kg, preventive group): recovery from cell necrosis, renal tubule damaged has been recovered, no glomeruli congestion. Slight edema and inflammation is still seen. (D) *Tephrosia purpurea* (200 mg/kg, curative group): recovery in necrosis was found. Peritubular congestion is still visible, tubular caste was also found. Renal tubular damage was recovered.

In the histopathological studies, the gentamicin-intoxicated group showed definite signs of nephrotoxicity, when compared to the control groups. The intoxicated group showed the presence of peritubular and glomerular congestion, tubular casts, epithelial degeneration, interstitial edema, blood vessel congestion, and infiltration by inflammatory cells, which are features of acute tubular necrosis observed in the histopathological sections and indicative of the extent of damage done at the tissue level, whereas the control group showed normal architecture. All the preventive and curative groups showed signs of recovery (Fig. 5).

## Discussion

In the various studies it has been reported that gentamicin activates phospholipases and alters the lysosomal membrane in addition to oxidative stress [10], although the mechanism of gentamicin-induced nephrotoxicity is not completely known. However, studies have implicated reactive oxygen species, particularly the superoxide anion radical, in the pathophysiology of gentamicin nephropathy [22]. It has been demonstrated that gentamicin administration increases renal cortical lipid peroxidation, renal nitric oxide generation, and mitochondrial H_2_O_2_ generation [23, 24]. Previously, *Aerva lenata* has been reported for its nephroprotective activity due to its free radical scavenging activity [25]. Natural antioxidants viz., ascorbic acid and alpha-tocopherol have also been found to be nephroprotective in an animal model [26]. Renoprotective effects have been reported for polyphenols such as quercetin [27]. Quercetin, one of the most abundant flavonoids, is a potent oxygen free radical scavenger and a metal chelator [28]. In addition, several studies indicate that quercetin inhibits the expression of COX-2 and iNOS (inducible nitric oxide synthesis) [29]. Thus, it is able to reduce the overproduction of nitric oxide (NO). NO has been reported as an oxidant in presence of superoxide anions. Both of these react and produce peroxynitrite, which is a potent and versatile oxidant [30]. It has already been demonstrated that there is an increased NO production during ischemia and gentamicin-induced renal failure [31]. Rutin also possesses potent iNOS-inhibiting activity which might protect against nitrosative kidney damage and may be a highly promising agent in preventing renal dysfunction due to ischemia/reperfusion injury [32]. *In vitro* experimental systems also showed that flavonoids possess anti-inflammatory properties [33].

The aqueous extract of *Cardiospermum helicacabum* and rutin were also reported to possess nephroprotective properties and the mechanism speculated for their protective effect is the ability to suppress the oxidative stress [34]. In another study carried out by Sharma et al. (2011), the effect of the ethanolic and aqueous extracts of *Bauhinia variegata* Linn. on gentamicin-induced nephrotoxicity in rats was studied. Although other constituents were also present along with rutin and quercetin, they were still discussed as major contributors to nephroprotection because of their antioxidant properties [35]. Singh et al. (2006) have also extensively reviewed the role of antioxidants in the prevention of renal diseases and rutin and quercetin were also reported as antioxidants and nephroprotectives [36].

Although a lot of studies have been carried out on *Tephrosia purpurea* (L.) Pers. (TP) for its pharmacological properties, the study regarding its renoprotective effects was lacking and the present study is a positive step in this direction. Our group also established the presence of flavonoids in TP and also reported the hepatoprotective activity of the ethanolic extract of TP in our previous studies [11]. Other groups have also reported the presence of some prenylated flavonoids [37]. The ethanolic extract of TP has been reported to possess wound healing activity in our previous studies [38]. Hepatoprotective activity was also reported in the aqueous-ethanolic extract of TP by Khatri et al. (2009) [39]. Much of the work has been done on the aerial parts of TP and the presence of rutin and quercetin has been well-documented in almost all of the studies, including the present study. Hence, the presence of these compounds in the leaves in the present study is justifiable and the observed activity can be attributed to their inherent antioxidant nature.

The ethanolic extract of TP leaves at a dose of 200 mg/kg significantly recovered the elevated blood urea and serum creatinine levels as compared to the control group and these findings are also supported by histopathological studies. The ethanolic extract has shown good superoxide radical scavenging, DPPH scavenging, and total antioxidant activity along with significant *in vivo* antioxidant activity. In the treated groups, GSH has been significantly increased, whereas the MDA level has been significantly reduced. GSH is the natural antioxidant present in the renal tissues. Hence, another probable mechanism of nephroprotective and curative activities of TP may be the ability to maintain GSH levels and inhibit the overproduction of NO and the inhibition of Cox-2 expression due to the presence of quercetin and other flavonoidal and phenolic compounds.

To conclude, our studies have shown that the leaves of TP possess marked nephroprotective and nephrocurative activities without any toxicity and thus, have a promising role in the treatment of acute renal injury induced by nephrotoxins, especially gentamicin. It is further concluded that the mechanisms involved are probably antioxidant activity and the inhibition of overproduction of NO and Cox-2 expression. We also conclude that the activities are attributed to phenolic and flavonoidal compounds, especially rutin and quercetin. However, the fact that the presence of the other reported flavonoidal compounds and phytoconstituents might be potentiating the effects of rutin and quercetin in the observed pharmacological activity cannot be ruled out.

## Figures and Tables

**Tab. 1:** Phytochemical screening of *Tephrosia purpurea* extracts.

Phytochemical constituents	Aqueous	Ethanol	Ethyl acetate	Chloroform
Carbohydrates	+	+	+	+
Alkaloid	−	+	+	+
Glycosides	+	+	+	+
Proteins	+	+	−	−
Phenolic compound	+	+	+	+
Fixed Oil	−	−	−	−
Volatile oil	−	+	−	−
Amino Acids	+	+	+	+
Flavonoids	+	+	+	+
Saponins	+	+	−	−

+…indicates presence and −…indicates absence of compound.

**Tab. 2:** Determination of blood urea and serum creatinine in serum of rats

Groups	Treatment regimen	Blood urea (mg/dl)	Serum creatinine level (mg/dl)
Group I	Control (Vehicle)	33.72 ± 1.92	0.818 ± 0.073
Group II	Gentamicin, (40 mg/kg, s.c.)	69.48 ± 4.34***	3.017 ± 0.208***
Group III (Preventive)	Ethanolic extract (200 mg/kg p.o.) + Gentamicin (40 mg/kg, s.c.)	45.44 ± 1.88**	1.84 ± 0.192***
Group IV (Curative)	Ethanolic extract (200 mg/kg p.o.) + Gentamicin (40 mg/kg, s.c.)	41.21 ± 2.28***	1.42 ± 0.122***

All the values are expressed as Mean± SEM. One way ANOVA followed by Dunnett’s test.

** P<0.01,

*** P<0.001 as compared to vehicle treated control group.

**Tab. 3:** Estimation of GSH and MDA

Groups	Treatment regimen	GSH (mg/100g tissue)	MDA (nmol/mg protein)
Group I	Control (vehicle)	45.6 ± 1.4	197.7 ± 2.1
Group II	Gentamicin (40 mg/kg, s.c.)	20.9 ± 3.2**	462.2 ± 4.3*
Group III (Preventive)	Ethanolic extract (200 mg/kg p.o.) + Gentamicin (40 mg/kg, s.c.)	31.2 ± 2.2**	264.8 ± 2.1*
Group IV (Curative)	Ethanolic extract (200 mg/kg p.o.) + Gentamicin (40 mg/kg, s.c.)	40.3 ± 2.9*	352.5 ± 4.9*

All the values are expressed as Mean± SEM. One way ANOVA followed by Dunnett’s test.

* P<0.05,

** P<0.01 as compared to vehicle treated control group.
